# Regional Reduction of Ganglion Cell Complex after Vitrectomy with Internal Limiting Membrane Peeling for Idiopathic Macular Hole

**DOI:** 10.1155/2014/372589

**Published:** 2014-11-16

**Authors:** Takayuki Baba, Eiju Sato, Toshiyuki Oshitari, Shuichi Yamamoto

**Affiliations:** Department of Ophthalmology and Visual Science, Chiba University Graduate School of Medicine, 1-8-1 Inohana, Chuo-ku, Chiba 260-0856, Japan

## Abstract

*Purpose*. To determine whether the reduction of ganglion cell complex (GCC) thickness is uniform in the parafoveal region after vitrectomy with internal limiting membrane (ILM) peeling for idiopathic macular hole (MH). *Methods*. Thirty-nine consecutive eyes were studied. Vitrectomy was performed with ILM peeling with brilliant blue G (BBG) staining, and room air was used for an intraocular tamponade. The GCC thickness and retinal sensitivity were measured at the superior, inferior, nasal, and temporal quadrants around the fovea using spectral domain-optical coherence tomography (SD-OCT) and microperimetry (MP-1). The measurements were made at baseline, and at 3 and 6 months postoperatively. *Results*. In 38 of the 39 eyes, the MH was closed after the initial surgery. At three and six months, the percentage of eyes with significantly thinner GCC areas was higher at the temporal quadrant (40.5% at 3 months and 46.0% at 6 months) than that at the other quadrants (*P* < 0.001, *P* < 0.001, resp.). The retinal sensitivity was also significantly lower in the temporal area than in the other areas (15.7 dB at 3 months, *P* = 0.003; 15.4 dB at 6 months, *P* = 0.006). *Conclusion*. These findings indicate that the inner retina in the temporal area may be more vulnerable to surgical manipulations than the other areas.

## 1. Introduction

The peeling of the internal limiting membrane (ILM) during vitrectomy has significantly improved the closure rate of macular holes (MHs) [1]. Because of this, ILM removal has become a common procedure during vitrectomy for MH repair. Another benefit of ILM peeling is the lower rate of reoperations due to unsuccessful closure resulting in a lower cost of treating an idiopathic MH compared to vitrectomy without ILM removal [2].

On the other hand, ILM peeling has been associated with alterations of the inner retinal surface, for example, a dissociated optic nerve fiber layer (DONFL) appearance [3, 4]. Originally, the DONFL appearance was believed not to affect retinal function [3, 5], but a more recent study by microperimetry showed a decrease in retinal sensitivity in the area of the DONFL appearance [6].

In addition, a reduction in the thicknesses of the inner retina and ganglion cell complex (GCC) has been reported after ILM removal during vitrectomy for a MH [7]. The thinning of the GCC was associated with a decrease in retinal sensitivity, and it was suggested that the thinning of GCC was caused by the ILM peeling rather than the toxicity of the dye used to make the ILM more visible [8].

A thinning of the GCC and reduction of retinal sensitivity have been reported by our laboratory. However, the results were based on measurements of the entire ILM peeled area, and the experiments were not designed to examine regional changes of the retina. In addition, the change in the thickness of macular area has been reported to continue for at least two years after ILM peeling, and the temporal retina keeps thinning but the other areas do not [9]. Therefore, the results of the present study are new because they showed a significant reduction of the GCC thickness in the temporal retina and a significant correlation between the regional thinning of the GCC and reduction of retinal sensitivity.

In some cases, our results showed that there were disagreements between the retinal sensitivity changes and GCC thickness. To try to resolve the lack of correspondence between the area of thinning of the GCC and the reduction of retinal sensitivity, we examined the regional changes of GCC thickness and retinal sensitivity after ILM peeling during vitrectomy for a MH. The pattern of thinning of inner retina was determined by OCT, and the associated reduction of the retinal sensitivity was determined by microperimetry.

## 2. Patients and Methods 

This was a retrospective, nonrandomized study of 39 eyes of 39 consecutive patients with an idiopathic MH. All cases were examined and treated at the Chiba University Hospital. This study was approved by Institutional Review Board of Chiba University Graduate School of Medicine, and the procedures used conformed to the tenets of the Declaration of Helsinki. Patients were informed on the purpose of the treatments and possible complications, and a written informed consent was obtained.

The surgery was performed by three of the authors (Takayuki Baba, Eiju Sato, and Shuichi Yamamoto) using similar procedures. Each of the three surgeons had more than 10 years of experience in performing vitrectomy for MH closure. A 3-port pars plana vitrectomy (PPV) with a 23-gauge system was performed with a combination of phacoemulsification and aspiration and implantation of a foldable intraocular lens if the eyes were phakic. A posterior vitreous detachment (PVD) was created if one was not present. Cases with an obvious epiretinal membrane (ERM), dense cataract (grade 3 or more by Emery-Little scale), glaucoma, and other retinal diseases were excluded.

The ILM was made more visible by 0.25% brilliant blue G (ILM blue, DORC, Zuidland, The Netherlands), and the ILM was gently grasped with an ILM forceps temporal to the fovea. In 6 eyes, the ILM peeling was started at the inferior retina to the fovea and these eyes were used as controls. The ILM was peeled off of the retina over approximately 3 disc diameters centered on the fovea. In all cases, an air tamponade was used and patients were instructed to maintain a face-down position for 3 days after the surgery.

All patients had a complete ophthalmic examination including measurements of the best-corrected visual acuity (BCVA), microperimetry (MP1, Nidek Technologies, Aichi, Japan), and spectral domain-optical coherence tomography (SD-OCT; RTVue-100, Optovue, Fremont, CA) before the surgery. Similar examinations were done at 3 and 6 months postoperatively. The data were collected and analyzed by an experienced examiner.

**Figure 1:**
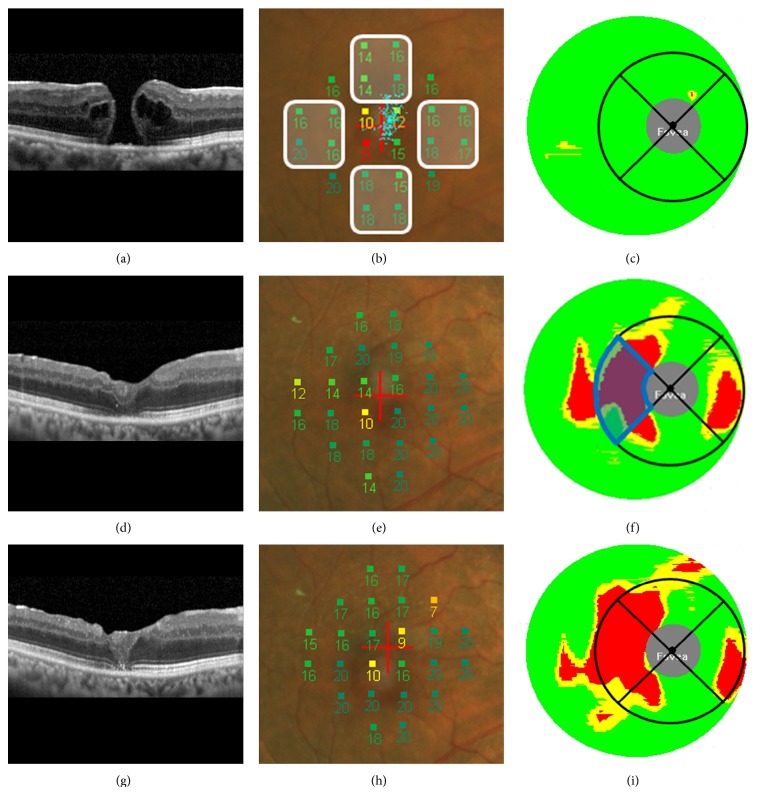
Right eye of a 67-year-old Japanese man with a macular hole (MH) treated by pars plana vitrectomy with ILM peeling. (a) Vertical OCT scan showing stage 3 MH. The preoperative decimal BCVA was 0.3. (b) Regional retinal sensitivity measured by MP1 was determined by averaging 4 locations shown in each white square. The sensitivity was 15.5 dB at the superior, 17.3 dB at the inferior, 17.0 dB at the temporal, and 16.8 dB at the nasal quadrants. (c) Ganglion cell complex (GCC) color-coded thickness map determined by SD-OCT of the preoperative eye. There are no red areas which would indicate that there are no areas thinner than that of the normative data. The diameter of the entire map is 6 mm and that of the inner ring is 4 mm. (d) Three months after the surgery, the horizontal OCT image shows that the MH is closed and the decimal BCVA has improved to 0.9. (e) Retinal sensitivity was 18.3 dB at superior, 18.0 dB at the inferior, 15.0 dB at the temporal, and 20.0 dB at the nasal area. (f) GCC thickness map 3 months after surgery. The percentage area of thin GCC (red) in one sector (blue) was calculated using software NIH ImageJ. In this case, it is 60.8% at the temporal quadrant. Similarly, the area of thin GCC was 14.8% at superior, 11.1% at inferior, and 26.9% at the nasal quadrant. (g) Six months after the surgery, the horizontal OCT showed MH closed, and the decimal BCVA was improved to 1.2. (h) The retinal sensitivity was 16.5 dB at the superior, 19.8 dB at the inferior, 16.8 dB at the temporal, and 20.0 dB at the nasal retina. (i) The percentage of thin GCC was 26.5% at the superior, 16.4% at the inferior, 83.5% at the temporal, and 21.2% at the inferior retina.

The retinal sensitivity was determined by MP1 with the 1.4.2.SP1 version of the software embedded in the device. The Goldmann III with a 4-2 staircase strategy was used and the sensitivity of 24 locations within the central 10 degrees was determined [10]. MP1 has an autotracking function and the retinal sensitivity was measured at exactly the same location of the fundus at each examination. The retinal sensitivity of the superior, inferior, nasal, and temporal areas surrounding the fovea was determined by averaging the sensitivity of four points in each area (Figure 1(b)). The retinal sensitivity at the central four points (central 2 degrees) was not included in determining the sensitivities of any of the quadrants.

The GCC thickness was determined by the GCC measuring mode of the original software of the SD-OCT. The GCC thicknesses are presented as color-coded significance maps obtained by comparing the findings of the patient to the normative database embedded in the computer. We evaluated the percentage of the total area of the GCC that was significantly thinner than the embedded database in each quadrant within the central 4 mm of the significance map (Figure 1(f)). The color-coded significance maps were displayed, and the areas shown by red were the significantly thinner areas. The number of red pixels in each area was counted by the NIH ImageJ software, and the number was divided by the total number of pixels in one quadrant. This percentage represented the significantly thinner regions in the quadrant, and these measurements were done for the superior, inferior, nasal, and temporal quadrants surrounding the fovea.

The outer retinal thickness was also analyzed using the software embedded in the SD-OCT. The average outer retinal thickness within the overall area is given automatically, and the baseline measurements were compared to the values at 3 and 6 months postoperatively.

Statistical analyses for the significance of differences of the GCC thickness and retinal sensitivity among the four quadrants were done with the Kruskal-Wallis test. The Mann-Whitney test was used to determine whether there were significant differences between temporal quadrant and other quadrants. The significance of the changes of the visual acuity was tested by Mann-Whitney *U* test. *P* values <0.05 were considered to be statistically significant.

## 3. Results

Twenty men and 19 women were studied, and their mean age was 66.7 ± 4.3 years with a range from 58 to 75 years. The estimated duration from onset of symptoms to surgery ranged from 1 to 8 months with a mean of 2.6 ± 1.9 months. The MH stage was stage 2 in 9 eyes, stage 3 in 20 eyes, and stage 4 in 10 eyes. The mean preoperative MH size was 638 ± 300 *μ*m and was the average of the vertical and horizontal diameters at the base of the hole. The baseline mean BCVA was 0.61 ± 0.33 logarithm of the minimum angle of resolution (logMAR) units (0.25 in decimal units) with a range from 0.15 to 1.22 logMAR units (0.06 to 0.7 in decimal units). The mean axial length was 23.5 ± 1.2 mm.

The MH was closed in 38 cases after the initial surgery, and the remaining eye was closed after a second vitrectomy. Data from this latter eye were excluded from further analyses. In 33 phakic eyes, phacovitrectomy was performed with an implantation of intraocular lens. No intraoperative or postoperative complications, including an elevation of the IOP, were observed.

**Figure 2:**
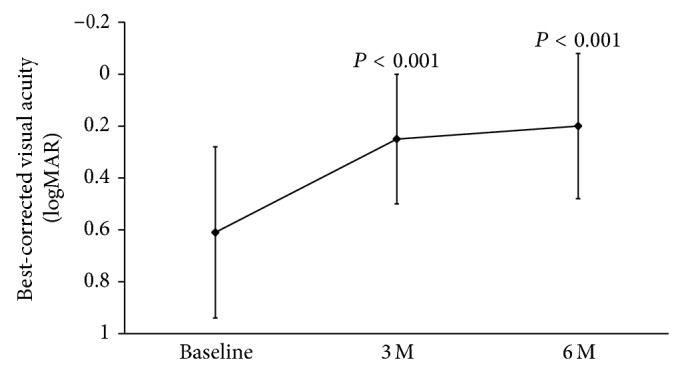
Changes of the best-corrected visual acuity (BCVA) up to 6 months after the MH surgery. BCVA is significantly improved after the initial surgery with ILM peeling at 3 and 6 months postoperatively (*P* < 0.001 and *P* < 0.001, Mann-Whitney test).

The findings in a representative case are presented in Figure 1. The mean preoperative BCVA was 0.61 ± 0.33 logMAR units (0.25 in decimal visual acuity), and it was 0.25 ± 0.30 logMAR units (0.56) at 3 months and 0.20 ± 0.28 logMAR units (0.63) at 6 months (Figure 2). The improvement in the BCVA from the baseline was significant at each time (*P* < 0.001 at 3 and at 6 months).

**Figure 3:**
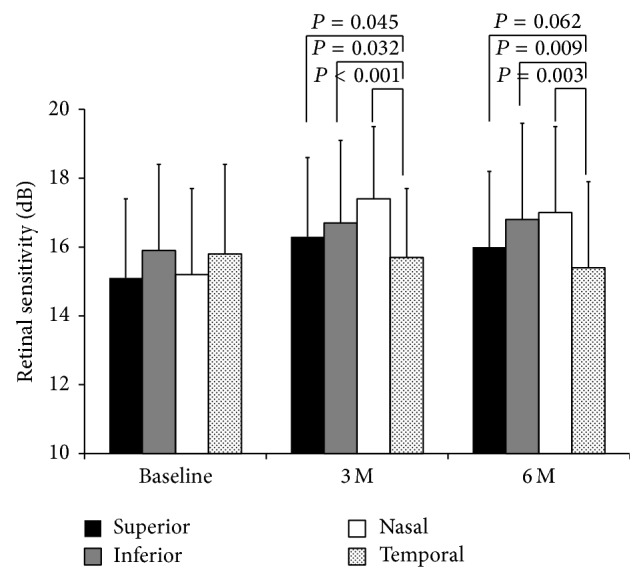
Regional changes of retinal sensitivity after the MH surgery. At 3 and 6 months postoperatively, the difference of retinal sensitivity between four quadrants was significant (*P* = 0.003 and *P* = 0.006, Kruskal-Wallis test). The retinal sensitivity at temporal quadrant was lower than those at other quadrants at 3 and 6 months (versus superior, *P* = 0.045 and *P* = 0.062; versus inferior, *P* = 0.032 and *P* = 0.009; versus nasal, *P* < 0.001 and *P* = 0.003, Mann-Whitney test).

At the baseline, the mean sensitivity at the four locations was 15.1 dB at the superior retina, 15.9 dB at the inferior retina, 15.2 dB at the nasal retina, and 15.8 dB at the temporal retina (Figure 3). None of these differences was significant. At 3 months after surgery, the retinal sensitivity was 16.3 dB at the superior retina, 16.7 dB at the inferior retina, 17.4 dB at the nasal retina, and 15.7 dB at the temporal retina. At 6 months, the retinal sensitivity was 16.0 dB at the superior, 16.8 dB at the inferior, 17.0 dB at the nasal, and 15.4 dB at the temporal retina. At 3 and 6 months postoperatively, the difference of retinal sensitivity between four quadrants was significant (*P* = 0.003 and *P* = 0.006). The retinal sensitivity at the temporal quadrant was lower than that at the other quadrants at 3 and 6 months (versus superior, *P* = 0.045 and *P* = 0.062; versus inferior, *P* = 0.032 and *P* = 0.009; versus nasal, *P* < 0.001 and *P* = 0.003). In the control eyes, the retinal sensitivity was 16.7 dB at the superior, 17.3 dB at the inferior, 17.5 dB at the nasal, and 16.1 dB at the temporal retina at 6 months postoperatively.

The average retinal sensitivity decreased from 15.6 dB to 15.4 dB at the temporal retina and changed from 15.3 dB to 16.5 dB at the other three quadrants in preoperatively phakic eyes. On the other hand, the average retinal sensitivity decreased from 16.7 dB to 14.7 dB at the temporal retina and changed from 15.8 dB to 16.4 dB at the other quadrants in the pseudophakic eyes.

**Figure 4:**
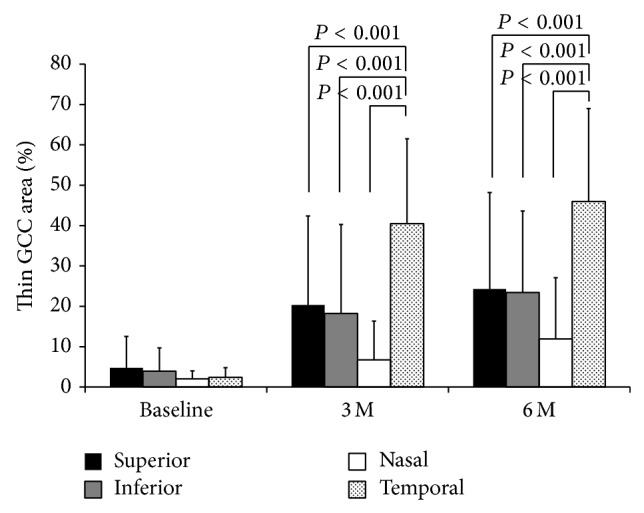
The percentage of thin GCC areas at the superior, inferior, temporal, and nasal quadrants. The difference of the percentage of thin GCC area was significant between quadrants at 3 and 6 months postoperatively (*P* < 0.001 and *P* < 0.001, Kruskal-Wallis test). The percentage of thin GCC area was significantly greater at the temporal quadrant at 3 and 6 months (versus superior, *P* < 0.001 and *P* < 0.001; versus inferior, *P* < 0.001 and *P* < 0.001; versus nasal, *P* < 0.001 and *P* < 0.001, Mann-Whitney test).

In our laboratory, the intraclass correlation coefficient of the measurements of thin GCC area is 0.917. The percentage of thin areas in the GCC was 4.7 ± 7.8% in the superior quadrant, 3.9 ± 5.8% in the inferior quadrant, 2.0 ± 7.5% in the nasal quadrant, and 2.4 ± 10.2% in the temporal quadrant at the baseline (Figure 4). The percentage of thin GCC areas became larger, and it was 20.3 ± 22.1% in the superior, 18.2 ± 22.1% in the inferior, 6.7 ± 9.6% in the nasal, and 40.5 ± 21.0% in the temporal quadrant at 3 months (*P* < 0.001, =0.001, =0.005, <0.001, resp., compared to the baseline). At 6 months, the percentage of thin areas of the GCC was 24.3 ± 23.9% in the superior, 23.4 ± 20.2% in the inferior, 11.9 ± 15.2% in the nasal, and 46.0 ± 23.0% in the temporal quadrant (*P* < 0.001, <0.001, =0.001, <0.001, resp., compared to the baseline). The difference of the percentage of thin GCC area was significant between quadrants at 3 and 6 monthspostoperatively (*P* < 0.001 and *P* < 0.001). The percentage of thin GCC area was significantly greater in the temporal quadrant at 3 and 6 months (versus superior, *P* < 0.001 and *P* < 0.001; versus inferior, *P* < 0.001 and *P* < 0.001; versus nasal, *P* < 0.001 and *P* < 0.001). In the control eyes, the percentage of thin areas of the GCC was 21.9 ± 24.9% in the superior, 24.6 ± 21.6% in the inferior, 9.9 ± 18.9% in the nasal, and 52.2 ± 23.4% in the temporal quadrant at 6 months. There was a trend towards more thinning of the GCC area at the temporal retina at 6 months postoperatively although the degree of thinning was not significant (*P* = 0.091). This was probably because of the small number of controls.

The mean outer retinal thickness was 178 ± 14 *μ*m at the baseline, 180 ± 13 *μ*m at 3 months, and 179 ± 12 *μ*m at 6 months postoperatively. There was no significant difference in the outer retinal thickness at 3 and 6 months compared to that at the baseline (*P* = 0.977 and *P* = 0.908).

There were no significant correlations between the temporal retinal sensitivity and MH duration (*P* = 0.140, *R* = 0.241), MH size (*P* = 0.912, *R* = 0.018), age (*P* = 0.784, *R* = 0.045), preoperative visual acuity (*P* = 0.088, *R* = 0.277), and axial length (*P* = 0.499, *R* = 0.111) at 6 months. There were no significant correlations between the temporal GCC thinned area and MH duration (*P* = 0.882, *R* = 0.027), MH size (*P* = 0.186, *R* = 0.240), age (*P* = 0.414, *R* = 0.150), preoperative visual acuity (*P* = 0.841, *R* = 0.037), and axial length (*P* = 0.460, *R* = 0.135) at 6 months.

## 4. Discussion

The retinal sensitivity measured by microperimetry was significantly lower at the temporal quadrant than at the other quadrants at 3 and 6 months after vitrectomy with ILM peeling for idiopathic MHs. In addition, the percentage of the GCCs that was significantly thinner than the embedded control values was significantly higher in the temporal quadrant at 3 and 6 months postoperatively. On the other hand, the outer retinal thickness measured by SD-OCT did not show any significant changes from the baseline.

A change in the appearance of the inner retina after the ILM peeling has been reported as a dissociated optic nerve fiber layer (DONFL) [3, 4, 11, 12]. The DONFL appearance consists of numerous arcuate striae within the area of the removed ILM. These striae were observed in blue-free fundus photographs [13] and in scanning laser ophthalmoscopic (SLO) images more easily than by standard ophthalmoscopy. TD-OCT analyses of the DONFL appearance found that it consisted of multiple defects of the nerve fiber layer [4, 12].

The normality of retinal function after the removal of the ILM remains controversial. The sensitivity of the retina in the area of the DONFL appearance has been reported to be not significantly different from the area without a DONFL [5, 13]. However, two recent studies reported a decrease in retinal sensitivity after ILM removal [6, 7]. The functional loss after ILM removal may partially be explained by observations of the SD-OCT images. The images showed that the changes at the area of the DONFL appearance were not limited to the nerve fiber layer but they extended into the ganglion cell layer [14] and into the inner plexiform layer in some cases [15, 16]. In this study, we found that the postoperative retinal sensitivity was lower at the temporal quadrant than at the other quadrants. On the other hand, the retinal sensitivity of the other three quadrants increased slightly mainly due to the removal of the cataract. The increase of retinal sensitivity at the other three quadrants was greater in the preoperatively phakic eyes than the pseudophakic eyes (1.2 versus 0.6 dB). In spite of the potential increase of retinal sensitivity by the cataract removal, the slight decrease of retinal sensitivity at the temporal retina (from 15.6 to 15.4 dB in phakic eyes; cf. from 16.7 to 14.7 dB in pseudophakic eyes) appears to be a significant loss of function. The area with low retinal sensitivity corresponded with the area of GCC thinning. These findings are consistent with earlier reports [16, 17] and suggest extensive retinal damage after ILM peeling, which is not limited to morphological changes but also to functional disturbances.

The reduction of the GCC thickness and retinal sensitivity after ILM peeling was most severe at the temporal quadrant. The reason for this restricted change to the temporal retina might be as follows. First, the removal of the ILM is started from the temporal retina to the fovea because we had assumed the temporal retina was safer to start the peeling of the ILM because the terminals of retinal nerve fibers exist at temporal retina. Although the surface of retina was gently grasped by forceps to try not to damage the retina, this might have affected the temporal GCC thickness. Second, the nerve fiber layer has been reported to be thinnest in temporal quadrant around fovea [18]. In addition, the density of ganglion cells at the temporal retina is less than that at the nasal retina within 2 mm from fovea [19]. The thinner nerve fiber layer and lower density of retinal ganglion cells may make the retina more vulnerable to mechanical damage. This idea is further supported by a study in which the deep defects of inner retina were observed at the temporal quadrant even if the initial ILM flap was created at the superior or inferior quadrants [16]. In our control eyes in which the ILM was peeled off from the inferior retina to fovea, the thinning of the GCC and reduction of retinal sensitivity were also at the temporal retina. Third, the macular region has been reported to be displaced toward optic disc after a closure of macular hole [20], which would mean that the temporal retina is stretched to become thinner. The relationship between the displacement of fovea and the thinning of temporal retina would be a point of interest and needs to be investigated in more detail.

BBG was used to make the ILM more visible. The ILM is transparent, and different agents including indocyanine green, trypan blue, BBG, autologous serum, and triamcinolone acetonide have been used to make it more visible. Some of these agents have been reported to be toxic to retinal neurons and can be responsible for the reduction of GCC. However, we suggest the influence of dye is minimal because BBG has been reported to be cytoprotective to retinal neuronal cells [21], and we have confirmed this by the good anatomical and functional recovery after MH surgery after BBG compared to indocyanine green [8]. We conclude that the mechanical damage of ILM peeling rather than the cytotoxicity of dye played a greater role in the thinning of the GCC postoperatively.

The major limitations of this study are the small number of eyes and the short postoperative observation period. The number of cases was relatively small because this study was conducted at a single institution. However, the surgical procedures were similar, and the collection of data was well organized. Three skilled surgeons with comparable experience of more than ten years performed surgeries. Although the ILM peeling area and its symmetry were not completely similar between cases, the peeling area was wide enough to cover the entire studied area. The observation period was six months and a longer postoperative period is needed because the thinning of the entire retinal thickness has been reported to continue for up to 24 months [9]. Even so, our data showed significant reduction of GCC thickness and retinal sensitivity and should be extended to additional eyes with longer follow-up periods.

In conclusion, we have investigated the regional changes of the GCC thickness and retinal sensitivity after ILM removal during surgery for an idiopathic MH. The findings that the GCC at the temporal quadrant is thinner and the retinal sensitivity at the same area was lower suggest that the inner retina at the temporal area may be more vulnerable to the surgical procedures.
